# The thoracoacromial axis in salvage head and neck reconstructive surgery, a case series

**DOI:** 10.1080/23320885.2022.2094270

**Published:** 2022-07-08

**Authors:** Matthew J. Davies, Rhys van der Rijt, Roger Haddad, James Southwell-Keely

**Affiliations:** a Department of Plastic and Reconstructive Surgery, St. Vincent’s Hospital Sydney, UNSW, Sydney, Australia; b Faculty of Medicine, UNSW, Sydney, Australia

**Keywords:** Thoracoacromial, reconstruction, head and neck, cancer, salvage

## Abstract

We present the surgical technique, relevant anatomy and a consecutive case series of salvage head and neck free flap reconstructions utilising the thoracoacromial axis. We demonstrated that the thoracoacromial axis is safe and reliable in salvage head and neck reconstruction with particular use in reconstruction of tracheoespophageal and pharyngolaryngectomy fistulae.

## Introduction

Free flap reconstruction in salvage head and neck surgery is a challenging undertaking. Following tumour excision, neck dissection, radiotherapy and previous free flap surgery, recipient vessels for salvage free flap microvascular anastomosis can be scarce. Healing is negatively impacted by patient factors, such as medical comorbidities and malnutrition. This situation demands recipient vessels to be of large calibre, consistent anatomy and easily accessible.

Various options for recipient vessels in this patient population have been reported including venous interposition grafts [1], cephalic vein transposition [2], arteriovenous loop procedures [3], thoracodorsal transposition [4], thyrocervical trunk [5], and using the pedicle of a previously transferred free flap [6].

The thoracoacromial axis provides a nearby recipient site as a potential option in the vessel depleted neck. We report six cases of free flap salvage in head and neck reconstruction in which the thoracoacromial axis was used as the recipient site for microvascular anastomosis, with the aim of assessing the safety and reliability of the thoracoacromial vessels in these challenging clinical situations.

## Surgical anatomy and technique for vessel preparation

**Figure 1.**
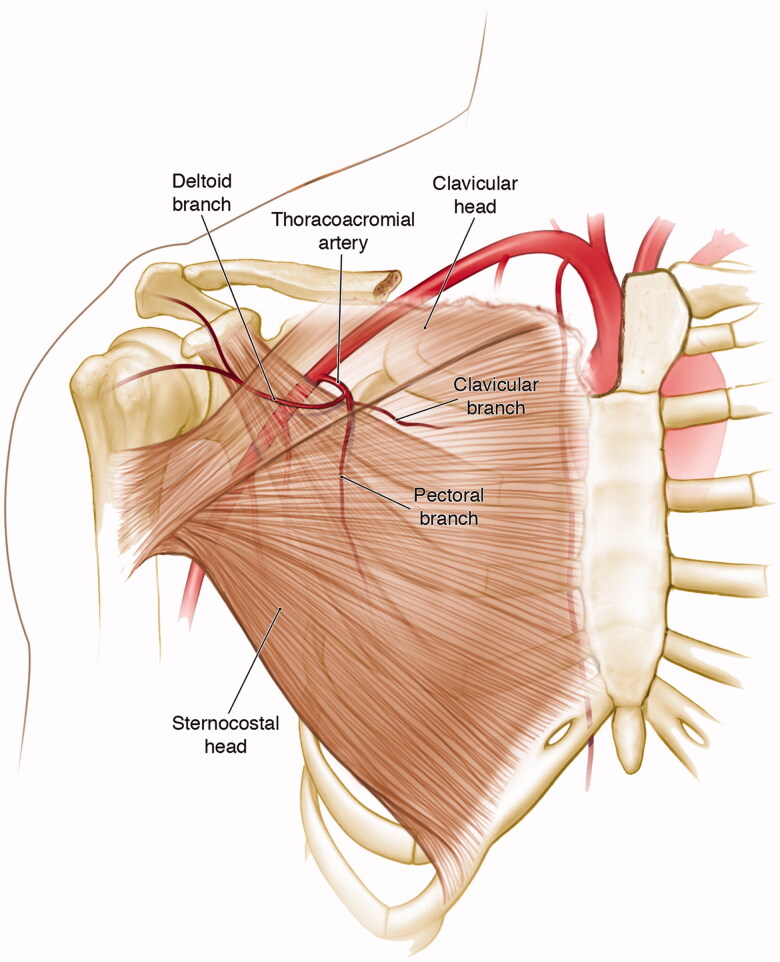
Schematic drawing of the thoracoacromial trunk arising off the second part (behind pectoralis minor) of the axillary artery and giving its deltoid, clavicular, pectoral and acromial (not pictured) branches. Adapted from Ref. [7].

The thoracoacromial artery arises from the second part axillary artery and divides into acromial, deltoid, clavicular, and pectoral branches. The pectoral branch, which supplies the pectoralis major muscle, penetrates the clavipectoral fascia 6–10 cm lateral to the sternoclavicular joint. It then runs along the deep surface of the pectoralis major muscle encased in a protective perivascular fatty tissue (Figure 1) [8].

**Figure 2.**
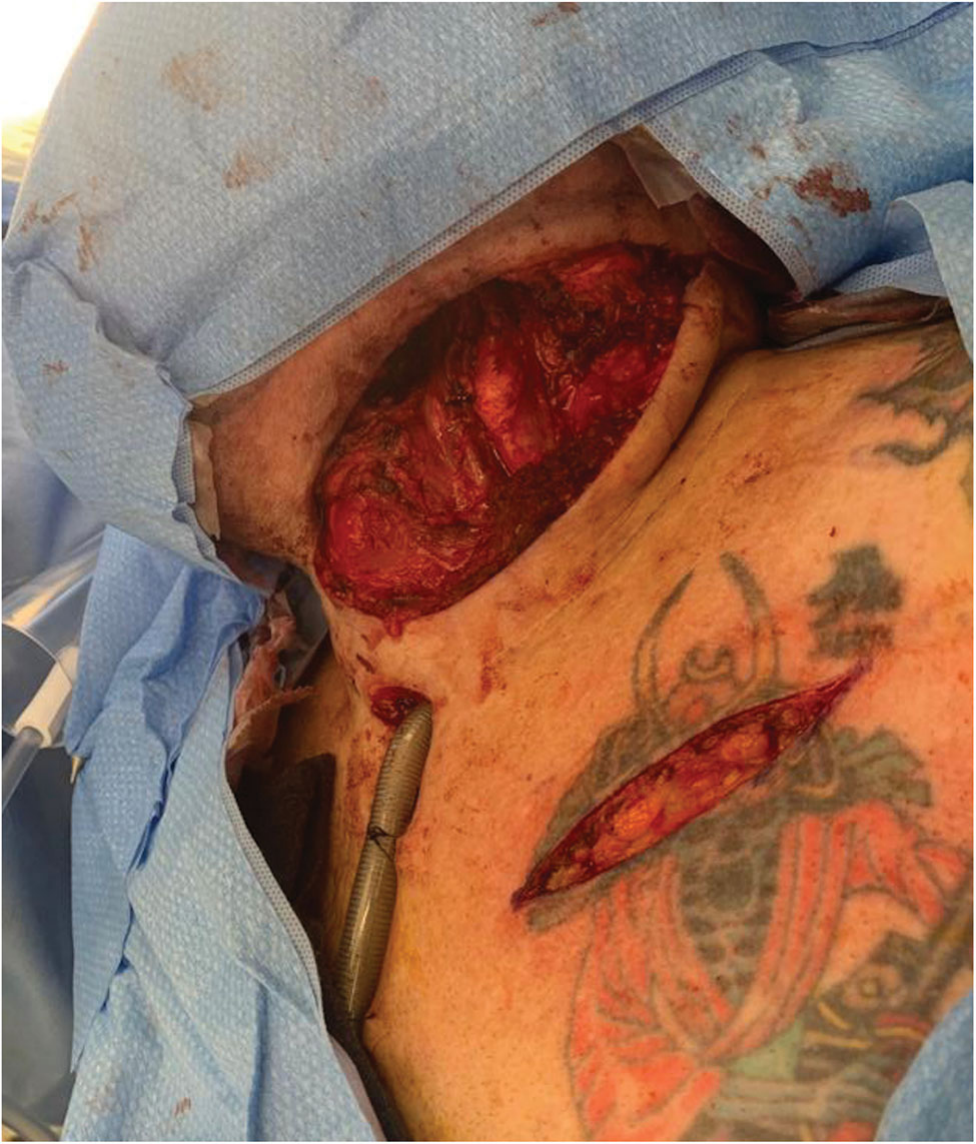
Intraoperative photograph demonstrating the defect and the infraclavicular incision, made 6–10 cm lateral to the sternoclavicular joint.

**Figure 3.**
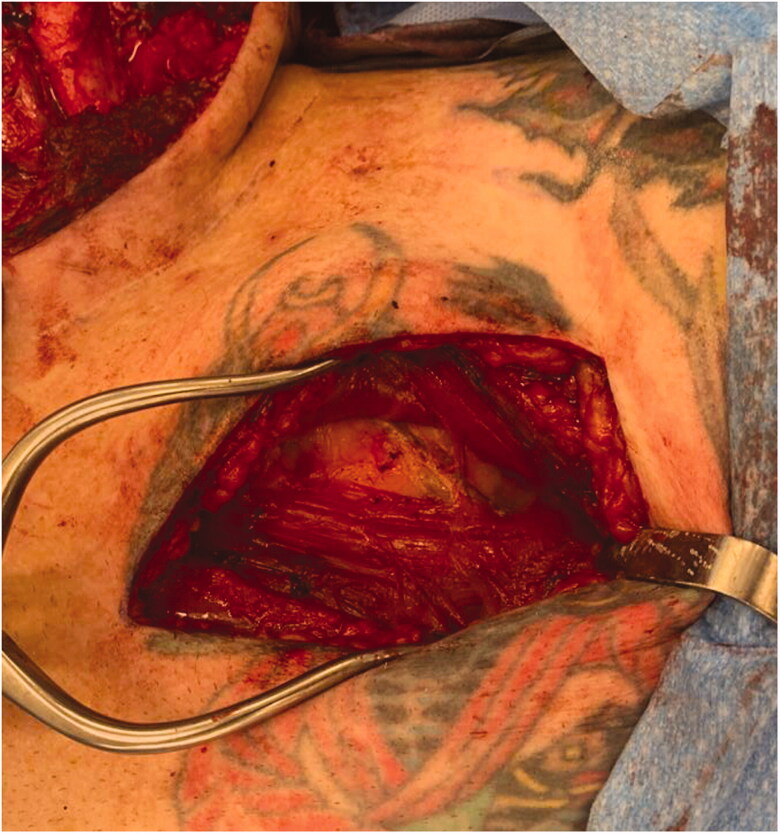
Intraoperative photograph demonstrating the pedicle, identified below the retracted clavicular and sternal heads of pectoralis major.

A 5 cm incision is made 2 finger breadths inferior to the clavicle, approximately 6–10 cm from the sternoclavicular joint. Rapid dissection down to the pectoralis muscle is then carried out with monopolar diathermy (Figure 2). The pectoralis major muscle is split between the clavicular and sternal heads and the perivascular fatty tissue is identified along with the pedicle (Figure 3).

## Patients and methods

We conducted a review of the free flap database at St Vincent’s Hospital in Sydney for the past 5 years, between 2016 and 2020, for patients in which the thoracoacromial axis had been used for salvage head and neck reconstruction recipient blood vessels. A retrospective review of case files was conducted. Data obtained included patient age, indications for surgery, prior free flap surgery, neck dissection and radiotherapy. Medical and surgical complication data including complete and partial flap loss was also collated.

## Results

From 2016 to 2020, a total of 6 patients underwent salvage free flap reconstruction following head and neck surgery utilising the thoracoacromial axis as the recipient vessels.

The average patient age was 66 and 5 of the patients were male. All six patients had prior radiotherapy and neck dissection with only one patient having a prior unilateral neck dissection only. All patients required salvage free flap reconstruction for tracheoesophageal or pharyngolaryngeal fistulae.

Fasciocutaneous flaps were used in five out of the six cases with one patient receiving a free gracilis muscle flap. In two out of the six cases, the clavicular branch of the thoracoacromial axis was used for arterial microvascular anastomosis. The microsurgical suture material used in all cases was 8.0 nylon which was employed in an end-to-end arterial anastomosis. All venous anastomoses were performed with the accompanying venae comitantes. Venous couplers were used for the venous microvascular anastomoses and their sizes ranged from 2.0 to 3.5 mm.

There were no flap complications including partial or complete flap loss. No cases required take back to theatre for collection or revision of the microvascular anastomoses. One patient experienced persistent pharyngeal fistula, which was successfully managed conservatively to complete healing and thereafter had an uneventful post-operative course. One patient developed a lower respiratory tract infection post operatively, which was treated successfully with intravenous antibiotics. There were no other medical complications of note. The mean follow up time was 15 months.

## Discussion

Finding recipient vessels for microvascular anastomosis in salvage head and neck free flap reconstruction is a challenging undertaking. In these situations, vessels are depleted secondary to cancer excision, neck dissection, and prior reconstructions. Furthermore, the operative field is hostile with post-surgical scarring and radiotherapy changes.

Free flap salvage head and neck surgery is a challenging endeavour. Recipient vessels must be of significant calibre and reliable. The ease of dissection and access for microsurgery is an additional benefit.

The thoracoacromial axis is consistently present and offers large calibre vessels for microvascular anastomosis. In all cases, the thoracoacromial artery and venae comitantes were of appropriate size for microvascular anastomosis. This recipient site has the added benefits of being easily accessible, the surgical time taken to prepare the vessels is minimal. In addition, the thoracoacromial axis is close to the operative field yet still out of the previous zone of surgery and radiotherapy.

We found the thoracoacromial axis particularly useful in reconstructing tracheoesophageal fistulae due to the proximity to the defect. In these situations, local flaps have high rates of breakdown and it is imperative to recruit well-vascularised tissue from outside of the previous surgical site and radiotherapy field.

Defect preparation is important. The fistula tract is excised, and necrotic tissue is debrided. A subcutaneous tunnel is prepared for the passage of the pedicle to the thoracoacromial vessels. In most instances, a folded radial forearm was used to patch the defect, with one part of the flap skin facing internally to reconstruct the lumen of the defect with the remaining flap being folded back on itself and to resurface the neck.

One potential drawback to consider when using the thoracoacromial axis for recipient vessels is that the pectoral branch supplies the pectoralis major muscle, a traditional lifeboat in head and neck cancer patients, however the blood vessels to the pectoralis major flap can be avoided by utilising one of the other branches of the thoracoacromial axis. Consideration needs to be given to the best reconstructive options for the salvage head and neck cancer patient, their general medical conditions and prior reconstructive attempts before planning any further reconstruction operations. The use of the thoracoacromial vessels for microvascular anastomosis following ipsilateral pedicled pectoralis major flap is also safe and has been well documented in the literature [9]. The pedicled pectoralis major flap has its own drawbacks, having limited reach and it is often bulky, which can be an issue when reconstructing a tracheoesophageal or pharyngolaryngectomy fistulae. However, the pedicled pectoralis major flap does retain utility in its reliability and ability to cover larger blood vessels especially post-radiation. With the advent of microsurgery, tailored free tissue transfer offers a wide variety of tissues options and ultimately leads to a superior reconstruction.

## Conclusion

The thoracoacromial axis is a reliable source of easily accessible recipient vessels in salvage head and neck surgery. In our unit, we have found it particularly useful in tracheoespophageal and pharyngolaryngectomy fistula repairs.
